# Nkx2.5: a crucial regulator of cardiac development, regeneration and diseases

**DOI:** 10.3389/fcvm.2023.1270951

**Published:** 2023-12-06

**Authors:** Ce Cao, Lei Li, Qian Zhang, Haoran Li, Ziyan Wang, Aoao Wang, Jianxun Liu

**Affiliations:** ^1^ Institute of Basic Medical Sciences of Xiyuan Hospital, China Academy of Chinese Medical Sciences, Beijing Key Laboratory of Chinese Materia Pharmacology, National Clinical Research Center of Traditional Chinese Medicine for Cardiovascular Diseases, Beijing, China; ^2^ Institute of Chinese Medicine Sciences, Guangdong Pharmaceutical University, Guangzhou, China

**Keywords:** transcription factor, Nkx2.5, myocardial regeneration, cardiac development, myocardium

## Abstract

Cardiomyocytes fail to regenerate after birth and respond to mitotic signals through cellular hypertrophy rather than cellular proliferation. Necrotic cardiomyocytes in the infarcted ventricular tissue are eventually replaced by fibroblasts, generating scar tissue. Cardiomyocyte loss causes localized systolic dysfunction. Therefore, achieving the regeneration of cardiomyocytes is of great significance for cardiac function and development. Heart development is a complex biological process. An integral cardiac developmental network plays a decisive role in the regeneration of cardiomyocytes. During this process, genetic epigenetic factors, transcription factors, signaling pathways and small RNAs are involved in regulating the developmental process of the heart. Cardiomyocyte-specific genes largely promote myocardial regeneration, among which the Nkx2.5 transcription factor is one of the earliest markers of cardiac progenitor cells, and the loss or overexpression of Nkx2.5 affects cardiac development and is a promising candidate factor. Nkx2.5 affects the development and function of the heart through its multiple functional domains. However, until now, the specific mechanism of Nkx2.5 in cardiac development and regeneration is not been fully understood. Therefore, this article will review the molecular structure, function and interaction regulation of Nkx2.5 to provide a new direction for cardiac development and the treatment of heart regeneration.

## 1. Introduction

Cardiomyocytes (CMs) fail to regenerate postnatally and respond to mitotic signals through cellular hypertrophy rather than cellular proliferation. Necrotic cardiomyocytes in the infarcted ventricular tissue are eventually replaced by fibroblasts, generating scar tissue, and the loss of cardiomyocytes causes localized systolic failure. Therefore, achieving the regeneration of cardiomyocytes is of great significance for cardiac function and development after the heart function is impaired. According to recent research, transplanted embryonic cardiomyocytes can minimize the amount of scarring in cardiac tissue and prevent post-infarct heart failure (1). As an innovative project, transplanting the cultured cardiomyocytes into the area of damaged myocardium is gradually being adopted. However, this innovative idea is extremely difficult to implement in a clinical setting due to the difficulty in obtaining ethical fetal hearts (2). Besides, cardiomyocyte-derived cell lines, which are extremely accessible and have fewer ethical issues, have the potential to replace fetal cardiomyocytes in this therapy. The field of regenerative medicine was revolutionized in 2006 by the discovery of induced pluripotent stem cells (iPSCs), which are cells reprogrammed into pluripotent stem cells by introducing a specific set of genes into adult cells, such as skin or blood cells (3). These iPSCs have the ability to differentiate into any cell type, including cardiomyocytes. The process of differentiating induced pluripotent stem cells (iPSCs) into cardiomyocytes involves several steps and can take several weeks. First, the iPSCs are induced to form mesodermal cells, which give rise to cardiac progenitor cells. These cells are then exposed to a combination of growth factors and small molecules that promote cardiac differentiation, resulting in Human induced pluripotent stem cell cardiomyocytes (hiPSC-CM) that have many of the properties of cardiac cells, including the ability to contract and respond to electrical signals (4, 5). Notably, the possibility of tumor formation by cardiomyocyte-derived cell lines is an urgent problem in clinical treatment. Therefore, exploring the specific mechanism of cardiomyocyte genesis and development is an important key to solving heart-related diseases.

The growth of a variety of muscle and non-muscle cell types is necessary for the intricate structure of the mammalian four-chambered heart. This includes the left and right atria's CMs, as well as the left and right ventricles, the conduction system, the pacemaker, the vascular smooth muscles, and the endo- and epicardial cells. Since cardiac development is a complex biological process, the complete cardiac development network is essential in the regeneration of cardiomyocytes (6). During this process, genetic epigenetic factors, transcription factors, signaling pathways, and small RNAs are involved in regulating the developmental process of the heart (7). Atrial natriuretic peptide (ANP), brain natriuretic peptide (BNP), myosin heavy chain (MHC), myosin light chain (MLC)-2a, MLC-2v, Nkx2.5, GATA-4, TEF-1, MEF2-A, MEF2-C, and MEF2-D are some of cardiomyocyte-selective genes that significantly aided myocardial regeneration (8, 9). Among them, Nkx2.5 is a key transcription factor associated with human congenital heart disease and one of the earliest markers of cardiac progenitor cells, so it is a promising candidate factor for the regeneration of cardiomyocytes (7). However, until now, the specific mechanism of Nkx2.5 in heart development and regeneration is not been fully understood.

We therefore will review the molecular structure, functional role, and interaction regulation of Nkx2.5 to provide new directions for myocardial regeneration and the treatment of heart disease. We regret any excellent research that has been left out or could not be covered owing to space restrictions.

## 2. Molecular structure of Nkx2.5

**Figure 1:**
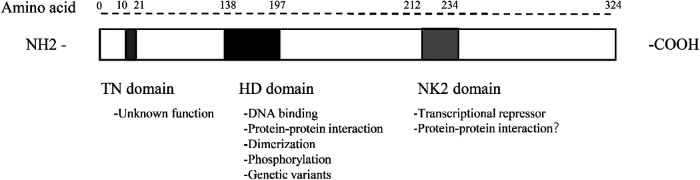
Protein structure and functional domains of Nkx2.5.

The Nkx2.5 gene is located at 5q35.1 of the human chromosome and is one of the NK2 members of the NK homologous box gene family. NK2 members include Nkx2.1, Nkx2.2, Nkx2.3, Nkx2.4, Nkx2.5, and Nkx2.6 (10). Alternative splicing mechanism enables cells to generate vast protein diversity from a limited number of genes by combining with different exons (11–14). The Nkx2.5 gene is 3,213 bp long, containing three exons that can form three isoforms through the alternative splicing mechanism (15). Isoform 1 contains an HD domain consisting of 324 amino acids, whereas isoforms 2 and 3 lack an HD domain (15). All three isoforms are expressed in fetal and adult cardiac tissues and are largely absent from extracardiac tissues by cardiac-specific RNA-sequencing studies and network analyses (7, 15). Nkx2.5 contains multiple structural domains from the N-terminal to the C-terminal, namely the TN domain (amino acids 10–21), the HD domain (amino acids 138–197), and the NK2 domain (amino acids 212–234) (Figure 1). The function of the TN domain remains unclear. The HD domain is the main functional domain of Nkx2.5 and determines the binding ability and transcriptional activation activity of Nkx2.5 to DNA. Its tertiary structure is a helix-turn-helix that specifically binds to the 5′T(C/T) AAGTG3′ sequence on DNA, with binding specificity determined by the tyrosine at position 54 in the third helix. Of a total of 1,380 unique genetic variants, 970 had their frequency information in the general population and 143 were associated with pathogenic phenotypes in humans. Furthermore, the homology domain has the greatest accumulation of pathogenic variants: 49 genetic variants in 60 residues and 23 genetic variants in the third *α*-helix, with 11 missense variants that could affect protein-DNA interactions or hydrophobic core (16). The NK-2-specific domain (NK2-SD) functions to mask the transcriptional activity *in vitro* reporter assays. The NK2-SD is proline-rich and may act as a protein-protein interaction interface.

## 3. Physiological function of Nkx2.5

Cardiac transcription factors are the major transcriptional activators of cardiac expression and regulate the expression of genes encoding cardiac-specific structural or regulatory proteins (17). The homologous cassette gene Nkx2.5 is the earliest transcription factor expressed in all vertebrate cardiogenesis and is involved in cardiac precursor cell differentiation, cardiac cyclization, atrial compartmentalization, atrioventricular outflow tract and conduction system formation (18, 19). Nkx2.5 genes play an important regulatory role in the complex cardiac developmental program and are critical for various physiological functions.

### 3.1. Marker of cardiac precursor cells

**Figure 2:**
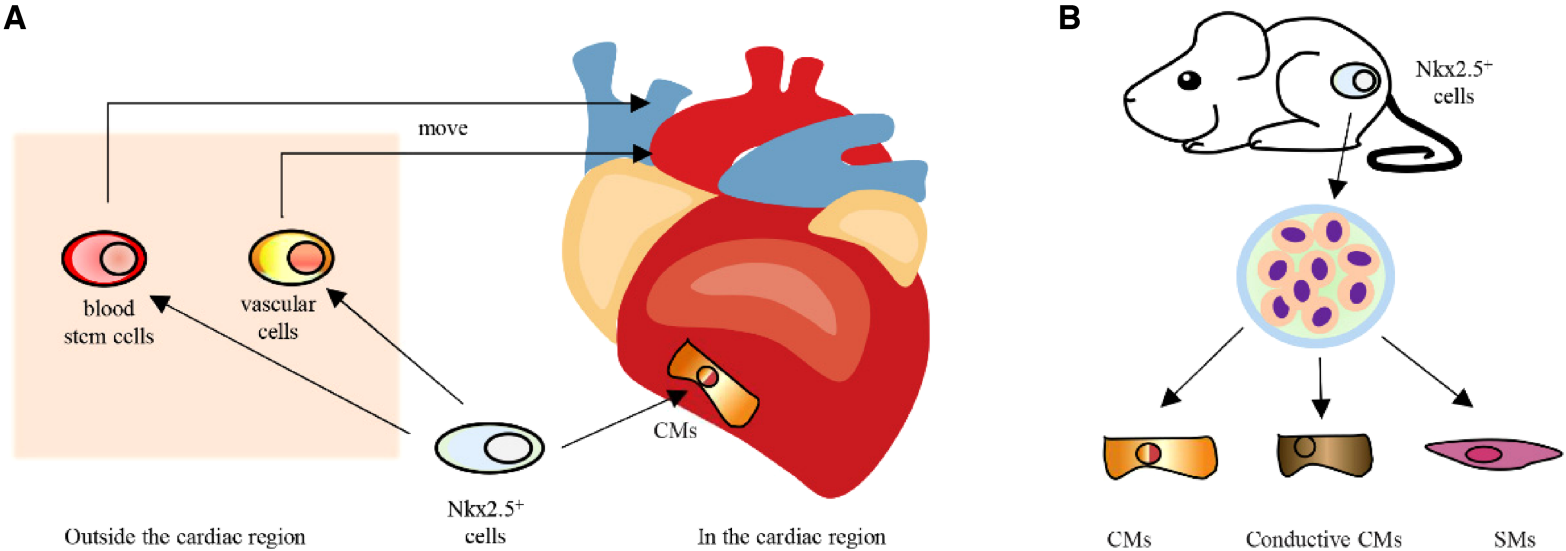
**A** Development of Nkx2.5 positive cells *in vivo*. (**B**) In vitro differentiation potential of Nkx2.5 positive cells. CMs, cardiomyocytes; SMs, smooth muscle cells.

Nkx2.5 was first expressed in embryonic cardiac precursor cells, much earlier than other cardiomyocyte markers, and can be consistently expressed during embryonic, fetal and adult stages (10, 20). In the cardiac region, Nkx2.5-positive cells will develop into cardiomyocytes. Outside the heart region, cells transiently expressing Nkx2.5 will move toward the heart site and develop into blood stem cells and vascular cells (21) (Figure 2A). In addition, when Nkx2.5-positive cells from mouse embryos are isolated and cultured *in vitro*, the majority of cells are found to develop into cardiomyocytes and related cells, which include conductive cardiomyocytes and smooth muscle cells (22, 23) (Figure 2B). According to the findings mentioned above, Nkx2.5 is strongly linked to the formation of the heart and can serve as a marker for cardiac precursor cells.

### 3.2. Regulation the development of the heart field

**Figure 3:**
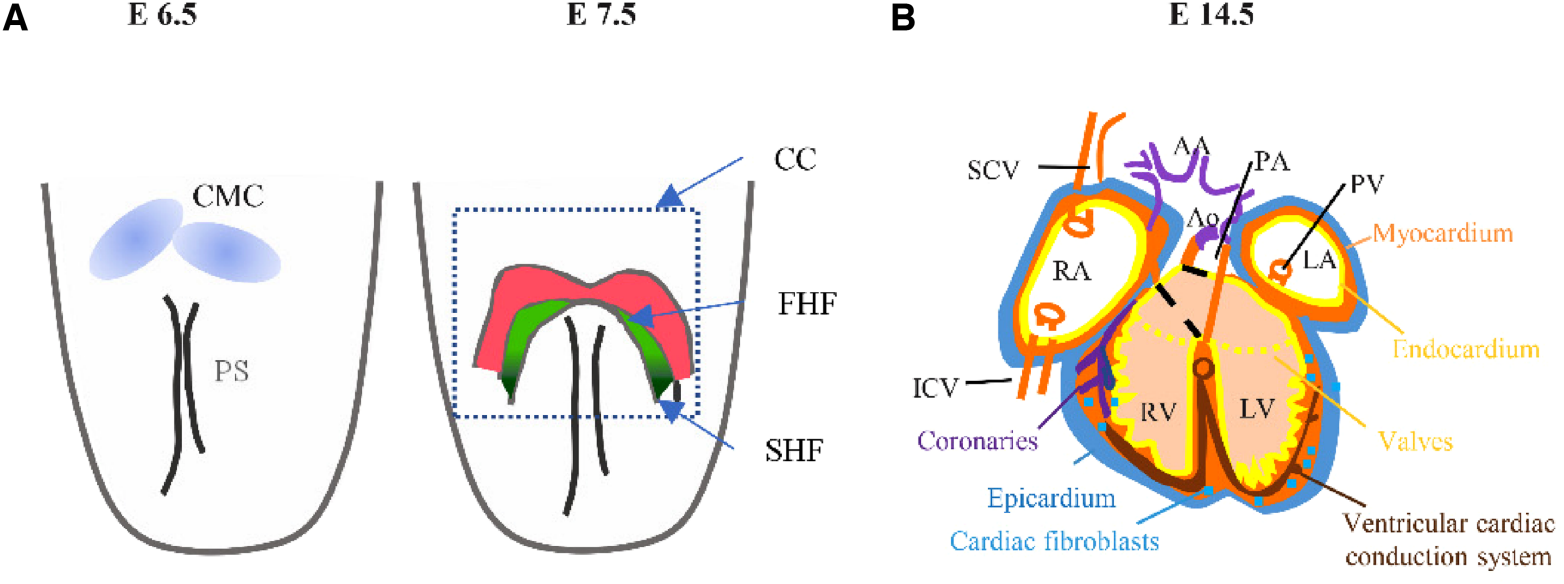
Schematic representation of cardiac mesodermal cells leaving the primitive streak (PS) (E6.5) contributing to the heart fields (E7.5) and to the tissues of the mature heart (E14.5), with color coding as in (**A,B**). E, embryonic day; AA, aortic arch arteries; Ao, aorta; CMs, cardiomyocytes; ECs, endothelial or endocardial cells; ICV, inferior caval vein; LA, left atrium; LSCV, left superior caval vein; LV, left ventricle; OFT, outflow tract; PA, pulmonary arteries; PEO, proepicardial organ; PV, pulmonary vein; RA; right atrium; RSCV, right superior caval vein; RV, right ventricle; SCV, superior caval vein; SMs, smooth muscle cells.

During embryonic development, the heart is the first solid organ that is capable of function. Mesodermal cells create the primitive streak during the earliest stages of embryonic development under the influence of secreted morphogens such as Wnts and BMP4 (24). The cardiac crescent, which gives birth to the primitive heart tube, is formed by the migration of cardiac mesodermal cells (CMC) in an anteromedial direction (25). Two heart-field-specific progenitors known as first heart field (FHF) and second heart field (SHF) are present in these migratory cells (26) (Figure 3A). FHF mainly differentiates into CMs and SHF participate in the elongation and looping of the heart tube.

According to the reporter system containing early cardiac transcription factors Nkx2.5 and TBX5, a study developed a human embryonic stem cell-derived line to isolate FHF and SHF. What's more, FHF CMs exhibit better sarcomeric structure and calcium handling, with closer location along the differentiation trajectory to human fetal hearts, due to the control of Nkx2.5 (27). CMs from first heart field may be a more suitable candidate for cardiac regeneration.

In the second cardiac region, Nkx2.5 can regulate the proliferation and differentiation of cardiac precursor cells to form intact and normal heart-related structures (28, 29). Nkx2.5 is expressed in the entire region of the embryonic heart, including the primitive left ventricular structure, the outflow tract stem formed by the development of the second ventricular region, the right ventricle, the atria and the venous sinus (30) (Figure 3B). Besides, there is a self-regulatory mechanism of Nkx2.5 expression in the second heart field, which plays an important role in maintaining the expression of Nkx2.5. Although this self-regulatory mechanism is evolution-conservative, there are some differences between mammals and non-mammals. In non-mammals such as chickens, Nkx2.5 binds directly to DNA enhancer regions to maintain Nkx2.5 expression, while in mammals such as mice, Nkx2.5 is indirectly self-regulated by Mef2c (31).

### 3.3. Regulation the development and functions of cells

In addition to the cardiomyocytes, there are myofibroblasts, vascular endothelial cells, Purkinje fibers and so on, which are also affected by Nkx2.5. In the process of wound healing and fibrosis, myofibroblasts are thought to arise *de novo* from fibroblasts and are characterized by induction of alpha-smooth muscle actin (alpha-SMA) expression, which is thought to be crucial to the abnormal deposition of collagen and propagation of fibrosis (32, 33). The result of research assays showed that Nkx2.5 could bind to both Nkx2.5 elements 1 (NKE1) and Nkx2.5 elements 3 (NKE3), which express in the alpha-SMA and inhibited alpha-SMA gene expression (34). By binding to NKE1 and NKE3, Nkx2.5 can regulate the alpha-SMA to affect myofibroblasts.

The expression of Nkx2.5 can be found in cardiovascular precursors (35). With the expression of Sry-type HMG box gene Sox17 from embryonic day (E) 7.5 to E8.5, Nkx2.5 cardiac progenitor cells specifically differentiate into the endocardium in mouse embryos (36). The pharyngeal arch arteries (PAAs) are temporary embryonic blood channels that contribute significantly to the carotid arteries and large cardiac vessels, such as the aorta and pulmonary artery. In the Nkx2.5 knockout zebrafish, the study displayed either a reduction in PAAs number or the absence of PAAs. Although Nkx2.5 is a key role of the endocardium, which is the endothelial component of the vertebrate heart, in heart regeneration and development, when, where and how the endocardium segregates during the process of embryogenesis have still remained largely unknown.

To start and spread electrical activity across the entire heart, the cardiac conduction system is crucial (37). Perfect synchronization between conduction and contraction, driven by specific conductive and contractile cardiomyocytes, is necessary for efficient heartbeats. After performing genetic fate mapping of Cx40^+^ cells, a study showed that Nkx2.5 dosage affects Purkinje fiber cells' (PFs) fate and network (38). Nkx2.5 expression requirements differ within various regions of the conduction system during development. Failure to express Nkx2.5 can result in hypoplasia of PFs, impacting the heart's ability to properly perform conduction system functions. This is critical knowledge for academics researching heart development and function. Besides, a maximum amount of Nkx2.5 is necessary during embryonic stages for appropriate Purkinje network formation, according to Cx40+ lineage tracing. However, what we need to pay attention to is that the expression level of Nkx2.5 only affects the differentiation of Purkinje fibers and has no effect on proliferation (39).

## 4. Transcription regulation of Nkx2.5

The embryonic development process begins with the formation of the heart, which is regulated by a group of cardiac core transcription factors. This important process is tightly controlled to ensure the proper development of this vital organ (40). Numerous previous studies have demonstrated that Nkx2.5 is regulated at the transcriptional level by many transcription factors and cis-regulatory elements upstream of the Nkx2.5. These transcriptions can regulate the expression of Nkx2.5 at different times and in different tissues (Table 1) (41).

**Table 1:** The gene regulatory network of Nkx2.5 controlling cardiac development.

Upstream regulatory factor	Synergistic regulatory factor	Downstream regulatory factor
MOG1 (42), Baf250a (43), Prox1 (45), Stat3 (46, 47), RHAU (48, 49), Bmp4 (50, 51), Shox (52, 53)	GATA4 (54–58), TBX5 (59–61), P53 (62, 63), Srf (64, 65), Smad4 (66, 67)	Furin (68, 69), CHD4 (70–72), GDF1 (73–75), R-spondin3 (76, 77), β-catenin (78, 79), Isl1 (80–82), HAND1 (84)

### 4.1. Transcription regulation of upstream target genes by Nkx2.5

Heart failure and cardiac hypertrophy were caused by the MOG1 deletion because it lowered the expression of Tbx5, which decreased the expression of Cryab and Hspb2 (42). What's more, silencing of mog1 expression leads to cardiac morphogenesis defects. To investigate the underlying molecular mechanism through which mog1 regulates cardiac development, the researcher employed whole-mount *in situ* hybridization to scrutinize the expression of key cardiac transcription factors in the anterior lateral plate mesoderm's heart-forming vicinity. The findings indicate that decreased expression of Nkx2.5 significantly contributes to the underdevelopment of cardiac structures following MOG1 knockdown (42).

The rhythmic beating of the heart depends on the sinoatrial node (SAN). Massively parallel sequencing analyses and chromatin immunoprecipitation coupled with quantitative real-time PCR results showed that Sinus bradycardia resulted from deletion of Baf250a in the SAN. Baf250a stimulates Tbx3 expression, while Baf250a, Tbx3 and histone deacetylase 3 work together to suppress Nkx2.5 expression (43). Due to the lack of Nkx2.5 gene expression, the function and development of cardiomyocytes are limited, which in turn leads to the abnormal conduction of the SAN, leading to bradycardia.

The transcription factor Nkx2.5 and the intermediate filament protein desmin are co-expressed in cardiac progenitor cells during the process of committed primitive mesoderm turning into the cardiomyogenic lineage. This suggests the importance of Nkx2.5 and desmin in the development of the heart. The lack of desmin expression can directly affect the transcription of Nkx2.5 gene in cardiac progenitor cells and thus affect cardiogenic commitment and myocardial differentiation. This situation was reversed with exogenous desmin treatment. Exogenous desmin stimulates transcription of Nkx2.5 reporter genes and rescues Nkx2.5 haploinsufficiency in cardiac progenitor cells. It also plays a crucial role in maintaining proper expression of Nkx2.5 in adult cardiac side population stem cells (44).

Through chromatin immunoprecipitation on adult hearts and gain and loss-of-function reporter assays *in vitro*, it was found that Prospero-related homeobox protein 1 (Prox1) plays a key role in adult cardiac conduction by directly repressing the function of Nkx2.5 via recruitment of the corepressor HDAC3. This repression takes place via a proximal upstream enhancer (45). These findings have important implications for understanding the mechanisms underlying adult cardiac function and may inform the development of new treatments for heart disease.

The signal transducer and activator of transcription (STAT) proteins are transcriptional regulators, responsible for mediating a host of biological functions in the body. Their primary function is to respond to signaling molecules found outside of cells and activate transcription accordingly. This makes them a critical component in a wide variety of physiological pathways (46). In a research endeavor, RNAi was utilized to knock down Stat3 within cells, and the expression of Nkx2.5 was analyzed. The findings demonstrated that during differentiation conditions, Nkx2.5 expression was reduced due to the elimination of Stat3. Conversely, the addition of exogenous Stat3 yielded a contrary outcome (47). Besides, the study also showed that Stat3 first induces the expression of Nkx2.5 and then together with Nkx2.5 induces the expression of GATA4 (47). These results suggest an intricate relationship between Stat3 and Nkx2.5 expression, emphasizing the importance of further investigations.

The G-quadruplex (G4) is a tetrad structure in both DNA and RNA molecules. It is formed by stacking G-quartets, which consist of four guanines arranged in a planar manner via Hoogsteen base pairing (48). The AU-rich element (RHAU) is an RNA helicase that can demarcate the G4 complexes (48). The 5′ non-coding region of Nkx2.5 mRNA contains a G4-chain structure that requires RHAU to melt, and an AU element in the 3′ non-coding region, which facilitates RHAU-mediated mRNA degradation (49). RHAU realizes the post-transcriptional regulation of Nkx2.5 by regulating the mRNA of Nkx2.5, affecting its stability and translation benefits.

The commitment of stem cells to differentiation, proliferation, and maturation is mediated by the transforming growth factor beta superfamily of cytokines, which includes bone morphogenetic protein 4 (BMP4) (50). Bmp4 is essential for the development of the embryonic heart. Heart patterning and looping are impacted by variations in Bmp4 localization. The absence of Bmp4 expression may result in the emergence of atypical heart architecture (51). Adipose-derived stem cells (ADSCs) are multipotent and have the capacity to differentiate into various cell lineages deriving from the mesoderm, ectoderm, and endoderm (50). According to the result of the study, Bmp4, which has established itself as one of the most effective differentiation factors for encouraging ADSC development into cells of mesodermal ancestry, via GATA4 controlled the expression of Nkx2.5 (51).

The development and differentiation of the sinoatrial node depend heavily on the early cardiac transcription factor short stature homeobox 2 (Shox2), which is the upstream transcriptional repressor of Nkx2.5. It has recently been proven that Nkx2.5 and Shox2 exhibit mutually exclusive expression patterns during the formation of SAN, as observed in academic research (52). Mutations in Shox2 affect the expression of Nkx2.5 in the sinoatrial node, resulting in fetal mortality in mice with severe sinoatrial node dysplasia and associated abnormal heart rate. Furthermore, a study has demonstrated that Shox2 initially restrains the expression of Nkx2.5 while stimulating the differentiation of pacemaker cells. This is then followed by the expression of Tbx3, HCN4 and Cx45. Shox2 overexpression has the effect of causing cells to differentiate into pacemaker-like cells rather than functioning as cardiac muscle cells (53).

### 4.2. Transcription regulation of synergistic target genes

GATA4 factors play crucial roles in the programming of cardiac cells during embryonic development (54). Although GATA4 is expressed in multiple cardiovascular lineages and Nkx2.5 is the marker of cardiac precursor cells, both of them are crucial transcription factors in gene regulation of cardiac development and cardiomyocyte differentiation (55, 56). Previous studies have demonstrated that the C-terminal zinc finger structure of GATA4 and the third helical structure of the Nkx2.5 HD domain (57). Furthermore, the results identify that GATA4 can work together with Nkx2.5 by specific residues within the GATA4s zinc finger domain and C-terminal extension (58).

TBX5 plays a key role in Holt-Oram syndrome, with a congenital heart defect (CHD) and atrial fibrillation being two of the most common cardiac phenotypes (59). According to research, TBX5 can bind to Nkx2.5 in the specific target DNA motifs through their centrally located DNA binding domains, the homeodomain (HD) in Nkx2.5 and TBox domain (TBD) in TBX5, during combinatorial interactions (60). Besides, the research also showed that in the Nkx2.5-HD/TBX5-TBD/DNA ternary complex structure, hydrogen bonds among conserved residues mediate the interactions between the proteins bound to the dual binding sites (60). By using an IDP that conforms to a rigid structure upon binding to a partner, TBX5 can interact with Nkx2.5, which can regulate the development of heart. What's more, through cotransfection of Nkx2.5 and Tbx5, it was found that the interaction between them synergically promoted the expression of downstream genes (61).

The P53 gene, a tumor suppressor that is found on human chromosome 17p13.1, produces the 53 kD intranuclear phosphorylated protein known as P53, which has 393 amino acids (62). Previous studies have shown that elevated P53 protein levels at critical phases of mouse development result in abnormalities in a variety of embryonic tissues, including the heart (63). The promoter of the striated muscle stress-responsive transcriptional cofactor Ankrd2, which controls the balance between proliferation and apoptosis during myogenic differentiation, is synergistically activated by P53 and Nkx2.5. Interestingly, the Nkx2.5 interaction site with P53 maps to the C terminal region, while P53 binding site on Nkx2.5 lies outside its C terminus (62).

The MADS-box transcription factor superfamily member SRF, which binds to the SRE region of the gene promoter and is involved in numerous crucial cellular processes including proliferation, differentiation, apoptosis, and growth cycle regulation, is crucial for embryonic development (64). The evidence supporting the cooperation between cardiac transcription factors Nkx2.5 and Srf is based on the interaction of Srf with a conserved binding site in the CpG island-like proximal promoter of Nkx2.5 (64, 65). This interaction is essential for the cardiac-specific expression, controlled by a SHF enhancer, and for the synergistic activation of these elements by cardiac transcription factors.

Smad4 is thought to be involved in cardiac development because disruption of the Smad4 gene resulted in the downregulation of cardiac-specific genes in Sfrp5-expressing cells, which are primarily cardiac progenitor cells for the FHF. That may account for the hypoplastic heart tubes seen in Smad4-knockout embryos (66). Smad4 can regulate the cardiac-specific gene expression and nuclear localization of Nkx2.5 (67). Following the activation of Bmp, the Smad1/5/8 complex binds with Smad4, which subsequently translocates into the nucleus to enhance the transcription of the Nkx2.5 gene. The Nkx2.5 protein is then phosphorylated by CK2, forming a protein complex with Smad4. This complex also moves into the nucleus and controls the transcription of the Nkx2.5 gene, effectively regulating the expression of Nkx2.5 (66). The Smad4 protein plays a vital role in cardiac development and myocardial regeneration through this mechanism.

### 4.3. Transcription regulation of downstream target genes

Furin is a secretory proprotein convertase (PC) that uses restricted proteolysis at one or more internal locations to convert precursor proteins into biologically active forms (68). Growth factors, transmembrane receptors, endocrine hormones, and adhesion molecules are only a few of the protein precursors that are thought to be matured by the PCs. Numerous possible Furin substrates have been connected to many facets of cardiac growth and differentiation. According to studies, the expression of cardiac Furin, which is essential for the maturation of cardiac progenitor cells and for the proper development of the atrioventricular junction and is expressed through modulating the bone morphogenetic proteins pathway, is directly repressed by Nkx2.5 (69).

One of the key chromatin remodeling complexes that mediate gene repression is the nucleosome remodeling and deacetylase (NuRD) complex (70). Heart development is one of the many developmental processes for which NuRD is crucial. Research, both clinical and genetic, has linked the catalytic subunit of NuRD, Chromodomain helicase DNA-binding protein 4 (CHD4), with congenital heart disease (CHD), specifically atrial and ventricular septal abnormalities (71). Furthermore, it has been proven that CHD4 is necessary for the development and operation of mammalian cardiomyocytes (72). Nkx2.5 enlists CHD4 to suppress noncardiac gene programs in the growing heart with the help of the nucleosome remodeling and deacetylase complex in order to ensure the normal development and regeneration of the heart (71).

Congenital heart disease is associated with genetic changes in the GDF1 coding area, and GDF1 is essential for left-right patterning (73). Vertebrates have dramatic left-right (L-R) asymmetries in the construction and location of their internal organs, a process known as left-right patterning, which marks the transition from symmetry to asymmetry (74). By binding to the promoter of GDF1, Nkx2.5 transactivated the expression of GDF1, which may confer genetic susceptibility to CHD potentially by altering its expression, according to the results of the luciferase assay, chromatin immunoprecipitation, and DNA pulldown assay (75).

R-spondins are powerful Wnt agonists and secreted factors that control stem cell growth to some extent (76). According to research, Nkx2.5 potentiates Wnt signaling by controlling the expression of the R-spondin3 (Rspo3) gene during cardiogenesis (77). Rspo3 is noticeably downregulated in Nkx2.5 mutants, indicating that Nkx2.5 is responsible for controlling Rspo3 expression (77).

As an adhesive connexin, β-Catenin, together with e-cadherin and alpha Catenin, forms an adhesive connexin complex, which maintains normal tissue structure and morphogenesis by regulating cell growth and intercellular adhesion. In addition, β-Catenin is a key functional effector molecule downstream of the classical Wnt pathway, which is crucial in embryonic development, tissue homeostasis, and tumorigenesis and development (78). By attaching to the unique components of the β-Catenin promoter region, Nkx2.5 can control the expression of -Catenin, which results in Nkx2.5 implementing regulation of the Wnt signaling pathway (79).

Isl1, a LIM homologous transcription factor, plays an important regulatory role in the survival, proliferation and migration of progenitor cells in the second cardiac region (80, 81). Besides, Isl1 can regulate the migration of progenitor cells to the original cardiac tube structure formed in the first cardiac region and promote its elongation and formation of cardiac structures (82). By performing Cre-loxP lineage tracing, the results showed that progenitors expressing both Nkx2.5 and Isl1 contribute to the proepicardium and express Wt1 and Tbx18, markers of epicardial progenitors, suggesting that Nkx2.5 and Isl1positive cardiac progenitors contribute to proepicardium formation during heart development (81, 83). What's more, it was discovered that Nkx2.5 binds directly to the enhancer region of Isi1 and suppresses the transcription of this gene, leading to aberrant myocardial development and regeneration (82).

HAND1, first heart field and early ventricular chamber marker, which is unique to humans, showed decreased expression in the CMs following Nkx2.5-knockout mouse, which is consistent with results from mouse and human CMs where Nkx2.5 is working upstream of HAND1 (84). It's interesting to note that the link between genes isn't always consistent. According to further findings, Nkx2.5 works upstream of HAND1 in cardiac mesoderm while HAND1 is upstream of Nkx2.5 in CMs (84).

## 5. Potential of Nkx2.5 in cardiac regeneration

**Figure 4:**
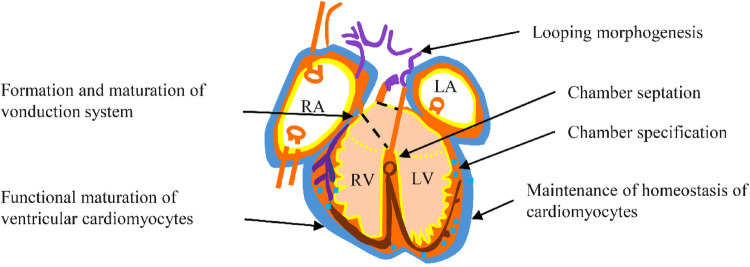
Functional role of Nkx2.5 during embyrogenesis, and in adulthood.

Nkx2.5 is crucial in regulating transcription and ensuring proper cardiac development and postnatal heart stability. It is particularly integral to cardiac morphogenesis, which involves rightward looping, followed by chamber specification and septation, as well as the functional maturity and upkeep of both the working myocardium and the conduction system (Figure 4).

Multiple tissues, including the heart, have been demonstrated to require parallel transcriptional networks for both development and regeneration. The stimulation of CM synthesis in injured myocardium depends on the reactivation of genes vital during cardiogenesis (85). The exogenous overexpression of Nkx2.5 in cardiomyocytes derived from ESCs resulted in the development of monocytes with characteristics of early ventricular cardiomyocytes, including the expression of sarcomeric markers, spontaneous beating frequency, and distinct occurrence of L-type calcium channels (86). These findings support the potential of Nkx2.5 overexpression as a strategy for inducing early cardiomyocyte differentiation.

The development of the heart tube and the adequacy of cell numbers are prerequisites for cardiac regeneration. Through inhibiting the function of the zebrafish Nkx2.5 gene, the research has revealed that Nkx2.5 plays an essential role in establishing the initial dimensions of the linear heart tube and the cell count. The absence of Nkx2.5 results in abnormal elongation of the heart tube, with the ventricular portion appearing unusually wide and short and the atrial portion exhibiting disorganization and sprawl. These findings highlight the crucial role of Nkx2.5 in heart development. Early in development, the ventricular cell count is normal at this stage, but the atrial phenotype is linked to an excess of atrial cardiomyocytes (87). However, later in development, when the heart chambers are forming, the number of ventricular cells is reduced in Nkx2.5-deficient embryos (87, 88). As a result, Nkx2.5 controls heart tube extension and has varied impacts on the amount of ventricular and atrial cells. These data indicate a late necessity for Nkx2.5 in chamber identity preservation based on the early expression of the Nkx2.5^−/−^ phenotype after cardiac tube development. Early Nkx2.5 activity is required for cardiac progenitor differentiation in order to preserve cellular characteristics and the architecture of the ventricular and atrial chambers later in development (89). As a result of linking an early need with a later function of chamber identity preservation, this newly established temporal connection broadens our understanding of the earliest responsibilities of Nkx2.5. Additionally, SHF cardiomyocytes have a later developmental need for Nkx2.5, highlighting the population's delayed specification and differentiation. Therefore, to maintain normal cardiac structural development, such as heart tube and cell number, it is essential to ensure continuous and stable expression of Nkx2.5.

Multivariate network analysis has identified Nkx2.5 as a critical regulator of cardiac muscle (CM) renewal, playing a crucial role in promoting cell cycle re-entry and maintaining genetic modules responsible for proteolysis (90). Ect2, psmb3, and psmd7 are direct targets of Nkx2.5 and serve as crucial hubs in the Nkx2.5-dependent gene regulatory networks (GRNs) that guide proliferation and sarcomere disassembly (91). Analysis of Nkx2.5^−/−^ fish has shown a reduced regenerative response following ventricular apex amputation due to compromised sarcomere differentiation and decreased proliferation, leading to inadequate regrowth of new CMs from pre-existing CMs (7). Furthermore, the failure of the epicardium to penetrate the regenerate hinders the establishment of the tissue environment necessary to induce a cascade of signals required for healing. It's interesting to note that in Nkx2.5^−/−^ embryos, early Nkx2.5 re-expression is sufficient to sustain a functioning cardiac rescue into maturity (89). The aforementioned text highlights the crucial role played by Nkx2.5 in the adult myocardium under stress conditions. This leads to the activation of regenerative signals, enabling the dedifferentiation and renewal of CMs and the establishment of a conducive microenvironment for effective patterning.

Ultimately, these data contribute to a better understanding of congenital heart disease and cardiac regeneration by shedding light on the processes that initiate and maintain chamber identity *in vivo*. These findings may contribute to the discovery of innovative approaches for purposeful differentiation of ventricular and atrial cardiomyocytes *in vitro*.

## 6. Association of Nkx2.5 mutations with heart disease

**Figure 5:**
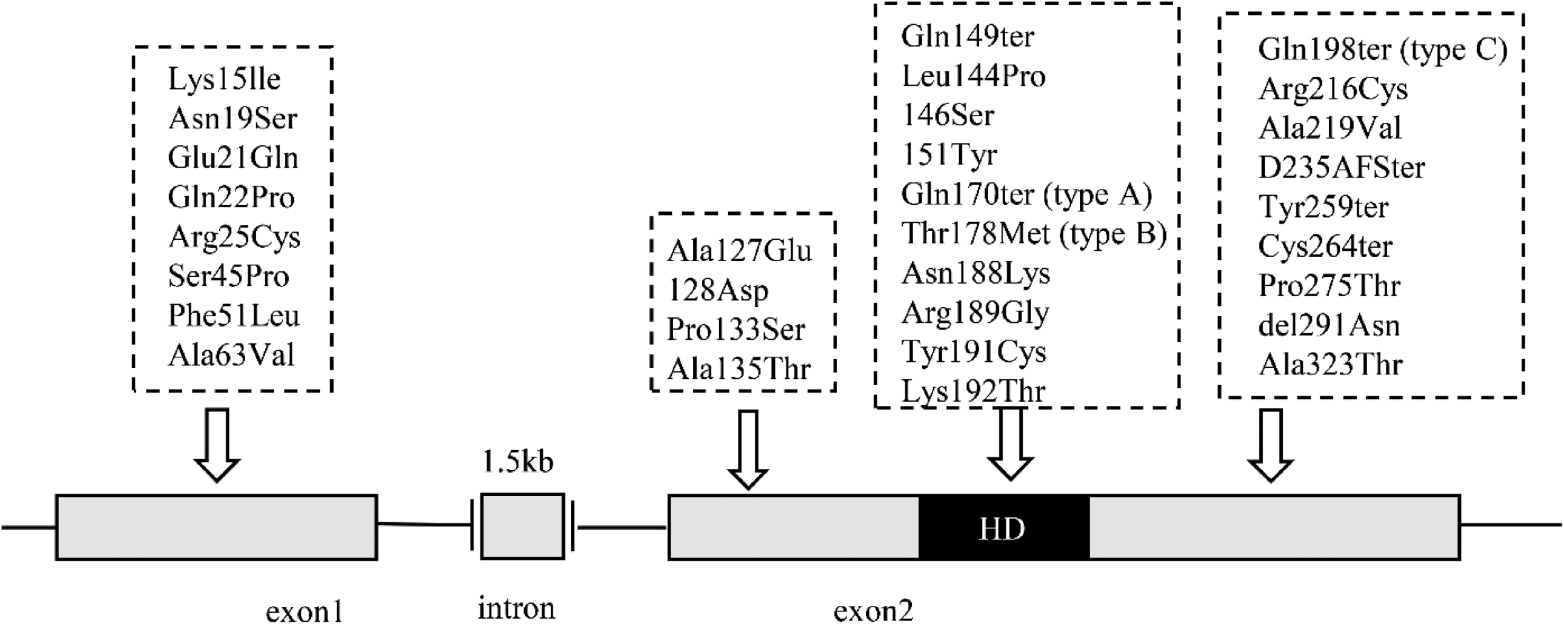
Schematic presentation of the CSX/NKX2–5 gene and the locations of the mutations. Two exons (open boxes) of Nkx2.5 are separated by a 1.5-kb intron. The homeodomain is denoted as a black box of Nkx2.5.

Since the Nkx2.5 mutation was first detected in CHD patients in 1998, researchers have been committed to exploring other disease-related sequence mutations in the NKx2.5 coding region (92–104) (Table 2). In afflicted individuals, the sequencing of the Nkx2.5 exons revealed three different types of mutations: one nonsense mutation (Gln170ter, type A), one missense mutation (Thr178Met, type B), and one nonsense mutation (Gln198ter, type C) placed C-terminal to the homeodomain (105) (Figure 5). Patients with a range of CHD have been shown to have over 10 disease-related mutations in CSX/Nkx2.5 thus far. The same type of CHD can have different Nkx2.5 gene mutations, and the same Nkx2.5 gene mutations can have different CHD phenotypes. Different heart malformations can even cause the same mutation, proving that modified genes and epigenetic effects cause different manifestations of the disease. The base deletion mutation was found to be located in the second helix of Nkx2.5 in 58 patients with CHD, which seriously affects the way and degree of DNA sequence binding to protein, leading to the uncontrolled transcription process, abnormal expression of downstream genes, and structural defects of related proteins, which lead to the occurrence of CHD (106). Nkx2.5 gene mutation is present in 3% of CHD patients, and these CHDs include atrial septal defect with abnormal ventricular septal defect, right ventricular double outlet, tricuspid valve malformation, tetralogy of Fallot, and vertebral trunk heart malformation (107). More than half of Nkx2.5 mutations are familial, and there are also specific abrupt inheritances in families. The research tested the Nkx2.5 gene of 230 patients with tetralogy of Fallot and found that the mutation frequency was 0.9% through genetic analysis (108). Nkx2.5 is the first cardiac transcription factor associated with the familial atrial septal defect, and 33 sudden changes have been found in familial atrial septal defect studies so far (109).

**Table 2:** List of Nkx2.5 mutations with heart disease.

Mutation				Heart disease	Reference
Regions	No.	Amino Acid Change	Nucleotide Change		
TN domain	1	Lys15Ile	A44T	ASD	(100)
	2	Asn19Ser	A232G	VSD	(96)
Homeodomain	1	Leu144Pro	T607C	ASD, AVSD	(96)
	2	146Ser	G614T	ASD, AVSD	(96)
	3	Gln149ter	C554T	ASD, VSD	(101)
	4	151Tyr	T629C	ASD, VSD, AVSD	(96)
	5	167Glu	A677C	VSD	(96)
	6	168Arg	C680T	VSD	(96)
	7	Gln170ter	C618T	ASD, AV Block	(103)
	8	176Lys	A704G	ASD, AVSD	(96)
	9	Thr178Met	C709T	VSD	(96)
	10	Thr178Met	C642T	ASD with HLHS	(104)
	11	Thr178Met	C533T	AV block, ASD	(104)
	12	181Gln	G543A	AVSD	(94)
	13	Lys183Glu	A723G	ASD, AVSD	(96)
	14	Trp185Leu	G554T	ASD, VSD	(95)
	15	Gln187Ter	C735T	VSD	(98)
	16	Asn188Lys	C673A	ASD	(101)
	17	Arg189Gly	C674G	ASD	(101)
	18	Arg190Cys	C568T	ASD	(104)
	19	Tyr191Cys	A681G	ASD, VSD	(101)
	20	Lys192Thr	A751C	VSD	(96)
	21	Lys192Arg	A751G	VSD	(96)
	22	Lys194Arg	A757G	VSD	(96)
	23	-	498–499insC	ASD, AV block	(95)
	24	-	605–606delTG	AV block	(95)
Nkx2.5 domain	1	Arg216Cys	C646T	Stenosis	(99)
	2	Ala219Val	C656T	Atresia, TOF	(97, 99)
	3	Asp226Asn	G852A	VSD	(98)
Other regions	1	Leu7Pro	T196C	AVSD	(96)
	2	21Glu	A239G		(96)
	3	Glu21Gln	G61C	TOF, Stenosis	(97, 99)
	4	Gln22Pro	A65C	TOF	(97)
	5	Glu22Arg	A65G	ASD	(102)
	6	Arg25Cys	C249T	VSD, TOF	(98, 101)
	7	Arg25Cys	C73T	Interrupted aortic arch, TA, TOF, HLHS, stenosis, atresia	(97, 99)
	8	Ser45Pro	T309C	VSD	(96)
	9	Phe51Leu	T327C	VSD	(96)
	10	Ala63Val	C188T	L-TGA	(97)
	11	Leu69Pro	T382C	VSD	(96)
	12	Pro77Leu	C406T	VSD	(96)
	13	Cys114Ser	T516A	ASD, AVSD	(96)
	14	Cys114Arg	T516C	ASD, VSD, AVSD	(96)
	15	Lys118Arg	A529G	ASD, VSD	(96)
	16	Ala119Ser	G355T	HLHS	(94)
	17	Ala119Glu	C356A	AVSD	(94)
	18	Lys124Arg	A547G	VSD	(96)
	19	Glu126Val	A553T	ASD, VSD, AVSD	(96)
	20	Ala127Glu	C380A	ASD	(97)
	21	128Asp	C560T	ASD, AVSD	(96)
	22	Pro133Ser	C573T	VSD	(96)
	23	Ala135Thr	G579A	ASD, ASD	(96)
	24	Gln198ter	C701T	ASD, AV block	(103)
	25	201Thr	T779C	VSD	(96)
	26	Val205Glu	T790A	VSD	(96)
	27	D235AFSter	InsTCCCT701	ASD, First-degress AV block	(97)
	28	242Gly	C902G	VSD	(96)
	29	Tyr248His	T918C	VSD	(96)
	30	Tyr259ter	C886A	ASD, VSD	(101)
	31	273Ala	T995C	ASD, VSD, AVSD	(96)
	32	Pro275Thr	C823A	CoA	(96)
	33	Ser279Pro	T1011C	VSD	(96)
	34	Ser279Phe	C1012T	VSD	(96)
	35	Ala286Val	C1018T	ASD, VSD, AVSD	(96)
	36	Ala286Val	C1033T	ASD, VSD, AVSD	(96)
	37	286Ala	C1034T	ASD, AVSD	(96)
	38	Del291Asn	delAAC871	DORV	(97)
	39	Asn294His	A1056C	ASD	(96)
	40	Asp299Gly	A1072G	ASD, VSD, AVSD	(96)
	41	301Asn	T1079C	VSD	(96)
	42	Ser305Gly	A1089G	VSD	(96)
	43	314Gly	A1118G	ASD, VSD, AVSD	(96)
	44	Gly320Ser	G1134A	ASD, VSD, AVSD	(96)
	45	Arg332Gln	G1141A	VSD	(96)
	46	322Arg	A1142G	ASD, VSD, AVSD	(104)
	47	Ala323Thr	G967A	TOF	(97)
	48	-	C113T	VSD	(104)
	49	-	C141T	VSD	(104)
	50	-	G1156A	ASD, VSD, AVSD	(104)
	51	-	215–221-delAGCTGGG	ASD, VSD, MV abnormality, Heterotaxia	(93)
	52	STOP-Gln	T1149C	ASD, VSD, AVSD	(99)
	53	-	Int1DSG+1T	AV block	(101)

RNA-seq profiling and dual-luciferase reporter assay revealed that the novel splicing mutation c.335–1G < A of Nkx2.5 can act upstream of PYK2 to regulate cardiomyocyte apoptosis via miR-19a and miR-19b (miR-19a/b) during the disease progression of atrial septal defects (ASD). Also, miR-19a/b is a downstream mediator of Nkx2.5 during cardiomyocyte proliferation (110). Thirty variants were detected in 439 Chinese patients with sporadic atrial septal defects, among which SNPs rs2277923 and rs3729753 were extremely prominent, with relatively high frequency and ratio in patients. Single nucleotide variants were the significant genetic variants of Nkx2.5 in Chinese patients with sporadic atrial septal defects (111). A study identified three variants of Nkx2.5: NM_004387.4:c.63A > G at exon 1, NM_004387.4:c.413G > A, and NM_004387.4:c.561G > C at exon 2 (112). The first variant is often observed (85.6%) and considered benign (112). However, the last two variants, located at the same locus, are infrequent (3.1%) and novel. Interestingly, these variants have been identified in familial cases of ASD, displaying a range of arrhythmias and severe pulmonary hypertension. Arrhythmia is an abnormality in the frequency, rhythm, origin, conduction velocity, or sequence of cardiac impulses. According to the principle of its occurrence, it can be divided into two categories: abnormal impulse formation and abnormal impulse conduction. Current studies have shown that Nkx2.5 gene mutations can cause defects in the conduction system of the human heart, leading to arrhythmias (113). Mutations in the Nkx2.5 gene, first identified in families with inherited atrioventricular block, which showed Ⅰ°–Ⅲ° atrioventricular block and so on, have been shown to cause a defect in the human heart's conduction system leading to arrhythmia (114). Mutations in Nkx2.5 are also found in patients with dilated ventricular arrhythmia, and may eventually lead to sudden cardiac death. Connexin 40 may be involved in arrhythmia. Related experiments have shown that the connexin 40 promoter has a binding site for Nkx2.5, and *in vivo* transcriptional activation studies have shown that Nkx2.5 can activate the expression of the connexin 40 promoter (115). What's more, mutations in the Nkx2.5 gene lead to apoptosis of autorhythmic cells, which may also be the cause of the arrhythmia (115). 15% of patients with sudden cardiac death have inherited atrial septal defect and atrioventricular block (116). Nkx2.5 mutations are present in 44% of families with atrial septal defects and sudden cardiac death (116). However, until now, there have been no relevant reports suggesting that the mutant allele is related to the severity of the phenotype (114).

In addition to these common malformations, other cardiovascular malformations caused by Nkx2.5 mutations have been reported. A study found that the p.K192X mutation in Nkx2.5 was thought to be associated with aortic valve malformation (117), and other research also reported the association of Nkx2.5 mutation with valve stenosis (118). What's more, the frameshift mutation of Nkx2.5 resulted in visceral inversus and sinus atrial septal defect (93).

## 7. Discussion

The regulation of the transcription factor Nkx2.5 is reliant on its various functional domains. Following transcription and translation, Nkx2.5 translocates to the nucleus and interacts with other transcription factors or binds to specific DNA sequences, consequently activating or inhibiting downstream regulatory molecules. As such, Nkx2.5 is a crucial player in modulating the proliferation, migration, differentiation, and function of myocardial precursor cells, while also maintaining the appropriate progression of cardiac development and the normative function of the cardiac conduction system. However, since Nkx2.5 is an important regulator of heart development, abnormal heart development and function are mostly caused by its dysfunction or gene mutation. The several functional domains of Nkx2.5 have a part in its regulatory function. However, as people age, their populations of cardiac progenitor cells become less plastic (119). Although a population of cells that reappears after myocardial infarction in the adult mouse heart is also recognized by the active cardiac-specific transcription factor Nkx2.5, the population of cells does not share the features predicted for embryonic myocardium (119). This suggests that only Nkx2.5 expression alone does not induce functional heart regeneration. Further studies revealed that transcription factors must be supported by a complete network of transcription factors for cardiac development.

Transcription factor networks, the cooperation between multiple transcription factors, are required for normal cardiac development and function. It is worth noting that our current understanding of the functional role of Nkx2.5 in the heart is limited, and further research is required to achieve a comprehensive understanding. Nkx2.5 is acknowledged as an early and specific indicator of cardiogenic profiles, and investigating the pathways that regulate Nkx2.5 expression will provide new insights into the molecular mechanisms behind the formation of the vertebrate heart. A study found that GATA, Nkx2.5, BMPs, and other transcription factors interact (120). In organisms with normal expression of this key transcription factor, Nkx2.5, when other family members are suddenly knocked out, it will obviously lead to more severe cardiac function or structural impairment. This suggests that a network of cooperative transcription factors is essential for cardiac function and structure (120).

The strict temporal sequence of expression of Nkx2.5 and other transcription factors such as BMP and GATA4 is one of the most important features in the evolution of the heart. Expression of the transcriptional network of Nkx2.5 and other factors is critical for heart development, however, expression of factors in the transcriptional network alone is not sufficient to support intact heart development. Only when each transcription factor in the transcription network is expressed strictly in accordance with the established time sequence, can each stage of the heart develop correctly and reasonably, and finally constitute a complete and healthy heart structure. In short, only when Nkx2.5, the transcription factor network, and the temporal expression are normal, can the heart have a chance to achieve normal structure, function and development. The comprehensive determination of concerted transcriptional regulation's significance reveals how interactions are regulated among various stages, cell types and extracellular stimuli. Moreover, the upstream and downstream framework of Nkx2.5 transcriptional regulation not only enhances understanding of the intrinsic cardiogenic program, but also provides valuable insights to further refine stem cell therapy. The discovery that stem or progenitor cells can differentiate into cardiomyocytes has prompted attempts by researchers to repair damaged hearts via the introduction of exogenous stem cells or stimulation of endogenous stem cells. However, due to the low efficiency of cardiomyocyte differentiation, these approaches have not yielded satisfactory results. A thorough comprehension of the transcriptional regulation of Nkx2.5 could optimize stem cell therapy efficacy by refining strategies aimed at encouraging stem cells to differentiate into highly specialized cardiomyocytes.

Only if all three items, Nkx2.5, the transcriptional network and the time sequence of the transcriptional network, are correct, then the heart develop into a normal functioning structure. Achieving all three conditions simultaneously is extremely demanding, and errors in any one of them can lead to heart-related diseases. Currently, most studies on Nkx2.5 have focused on a single pathway or a single time, with fewer studies on the overall transcriptional network and time course, which has led to an incomplete understanding of Nkx2.5, a key transcription factor in the heart. This requires that we adopt more advanced technologies, such as single-cell technology and spatial transcriptomes, which allow us to construct a complete, correct, time-course Nkx2.5 expression profile. Future studies should focus on the construction of complete transcriptional networks and the dynamics of transcriptional processes.

In summary, Nkx2.5 can influence cardiac precursor cells, cardiomyocytes, cardiac endothelial cells, fibroblasts, and Purkinje cells through interaction with other transcription factors or molecules, including transcription, translation, and into the nucleus, and ultimately regulate cardiac development and heart disease from multiple perspectives.
